# Non-Amide Polymers as Kinetic Hydrate Inhibitors—Maleic Acid/Alkyl Acrylate Copolymers and the Effect of pH on Performance

**DOI:** 10.1021/acsomega.1c06063

**Published:** 2021-12-29

**Authors:** Janronel Pomicpic, Radhakanta Ghosh, Malcolm A. Kelland

**Affiliations:** Department of Chemistry, Bioscience and Environmental Engineering, Faculty of Science and Technology, University of Stavanger, N-4036 Stavanger, Norway

## Abstract

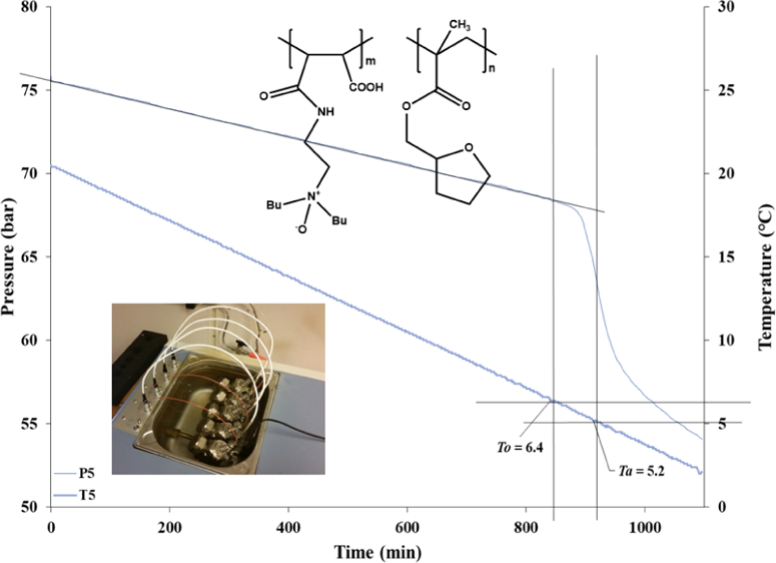

Kinetic hydrate inhibitors (KHIs) have been used for over 25 years to prevent gas hydrate formation in oil and gas production flow lines. The main component in KHI formulations is a water-soluble polymer with many amphiphilic groups, usually made up of amide groups and adjacent hydrophobic groups with 3–6 carbon atoms. KHI polymers are one of the most expensive oilfield production chemicals. Therefore, methods to make cheaper but effective KHIs could improve the range of applications. Continuing earlier work from our group with maleic-based polymers, here, we explore maleic acid/alkyl acrylate copolymers as potential low-cost KHIs. Performance experiments were conducted under high pressure with a structure II-forming natural gas mixture in steel rocking cells using the slow (1 °C/h) constant cooling test method. Under typical pipeline conditions of pH (4–6), the performance of the maleic acid/alkyl acrylate copolymers (alkyl = *iso*-propyl, *iso*-butyl, *n*-butyl, tetrahydrofurfuryl, and cyclohexyl) was poor. However, good performance was observed at very high pH (13–14) due to the thermodynamic effect from added salts in the aqueous phase and the removal of CO_2_ from the gas phase. A methyl maleamide/*n*-butyl acrylate copolymer gave very poor performance, giving evidence that direct bonding of the hydrophilic amide and C4 hydrophobic groups is needed for good KHI performance. Reaction of the maleic anhydride (MA) units in MA/alkyl acrylate 1:1 copolymers with dibutylaminopropylamine or dibutylaminoethanol gave polymers with good KHI performance, with MA/tetrahydrofurfuryl methacrylate being the best. Oxidation of the pendant dibutylamino groups to amine oxide groups improved the performance further, better than poly(*N*-vinyl caprolactam).

## Introduction

Kinetic hydrate inhibitors (KHIs) have been used since the mid 1990s to prevent gas hydrate blockages in oil and gas production flow lines.^1−6^ The potential formation of these ice-like solids of clathrate small hydrocarbons constitutes one of the most significant flow assurance issues for offshore fields.^7−10^ KHIs are formulations of one or more structurally specific water-soluble polymers in solvents and other synergists. KHIs can affect both the gas hydrate nucleation and crystal growth processes, delaying the build-up of gas hydrates for a length of time depending on the driving force (chemical potential) of the system.^11,12^ KHIs have been shown to both increase the nucleation work required to form critical size nuclei and increase the effective number of sites where nucleation could occur.^13^ The driving force of the system is often described in terms of subcooling, but other factors including the absolute pressure must be taken into account.^14−18^ There is evidence that KHIs can give total inhibition for an indefinite period up to a certain driving force.^19^

**Figure 1:**
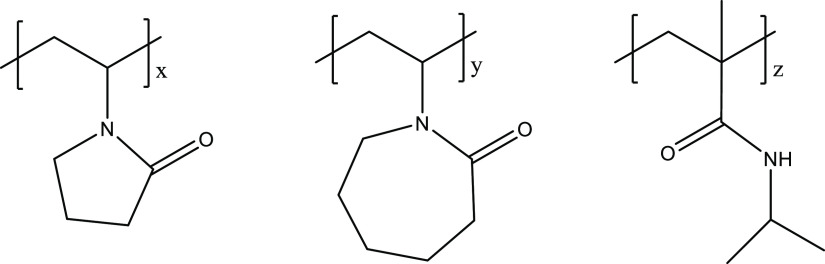
Industrially deployed KHI polymers. Left to right: PVP, PVCap, and PNIPMAM.

KHIs are injected into the produced well stream at concentrations of about 1–5 wt % in which the polymer usually makes up about 10–20% of the formulation. Most, if not all, commercial KHI polymers are amide-based polymers, such as poly (*N*-vinyl pyrrolidone) (PVP), poly (*N*-vinyl caprolactam) (PVCap), poly (*N*-*iso*-propyl methacrylamide) (PNIPMAM), and copolymers thereof (Figure 1).^20^ Developing more efficient and cheaper KHIs is still a goal that needs to be fulfilled.

**Figure 2:**
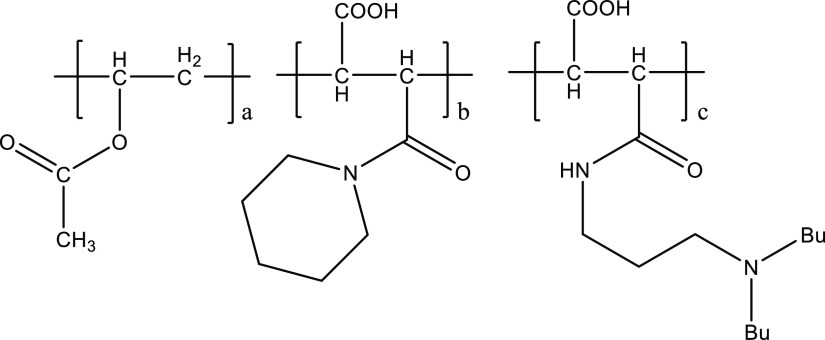
Product from the reaction of the vinyl acetate/MA copolymer with cyclohexylamine and 3-di-*n*-butylaminopropylamine (VA:MA-60%cHex-40%DBAPA).

Recently, we have explored polymers without amide groups as KHIs and shown that some polymer classes can give reasonable performance, albeit not as powerful as the best amide-based polymers.^21−24^ We have also investigated maleic-based amide polymers as KHIs since the monomer maleic anhydride (MA) is a cheap raw material. Maleic-based KHI polymers have been known since the 1990s, but recent advances in structure–activity analysis have led to polymers with improved performance.^25,26^ For example, the vinyl acetate/MA copolymer in which the anhydride is reacted with a 60:40 mixture of cyclohexylamine/3-di-*n*-butylaminopropylamine [VA:MA-60%cHex-40%DBAPA, *M*_n_ = 11 kg/mol, 25 wt % in 2-butoxyethanol (nBGE)] gave a significantly better performance than previously reported maleamide polymers (also in nBGE) or PVCap (Figure 2). Of course, the optimal KHI polymer will vary somewhat depending on the field conditions, but based on the jump in performance from our screening tests, it is likely that VA:MA-60%cHex-40%DBAPA will be a significant improvement in maleic polymers for a range of field conditions. This copolymer also demonstrated excellent compatibility at high temperatures, giving no cloud point in water at 95 °C as a 1.0 wt % solution.

As an extension of our work on non-amide KHI polymers, here, we explore non-amide derivatives of maleic copolymers by using widely available alkyl acrylates as the comonomers. The goal was to develop even more effective KHIs in which all the comonomers and solvents are more cost-effective raw materials than known KHI monomers such as VP, VCap, or NIPMAm. We have investigated the new alkyl acrylate/maleic copolymers as gas hydrate KHIs by using our standard natural gas (SNG) mixture and at varying pH.

## Experimental Section

### Materials

MA (≥99%), xylene (99%), 1,2-dimethoxyethane (DME, 99%), 2-butoxyethanol (nBGE, 99%), isopropylmethacrylate (*i*PrMA), *n*-butyl methacrylate (*n*BuMA), *n*-butyl acrylate (*n*BuA), *iso*-butyl acrylate (*i*BuA), tetrahydrofuran methacrylate (THFMA), cyclohexyl methacrylate (CHMA), and all amines were purchased from VWR (Avantor) and used as received. PVCap (MW, approximately 2–4 kg/mol) was supplied from BASF as Luvicap EG, a 41.1 wt % solution of the polymer in monoethyleneglycol. The solvent was removed for this study by repeated precipitation of the polymer from the aqueous solution above the cloud and deposition point (ca. 40 °C). Synthesis of polymaleic anhydrides was carried out according to the literature, except that toluene was replaced by xylene or DME. MA/alkyl methacrylate copolymers were made in the same way using azobisisobutyronitrile (AIBN) initiator and DME as the solvent.

### Synthesis of MA/Alkyl Acrylate Copolymers

An example of synthesis is given here for the MA/THFMA 1:1 copolymer: MA (0.89 g, 0.0091 mol), tetrahydrofurfuryl methacrylate (1.55 g, 0.0091 mol), AIBN (0.15 g, 0.91 mmol), DME (30 mL), and a stirrer bar were added to an ampoule. The air from the ampoule was then removed using a vacuum pump, and the ampoule was covered with a rubber septum. A syringe with a balloon was then attached. A stream of nitrogen gas was then flushed carefully through the ampoule. The reaction mixture was then heated and stirred at 70 °C on an oil bath, taking care not to overheat the bath to avoid too rapid decomposition of the AIBN initiator. After 15 h, the system was cooled to room temperature, and the solvent was removed at reduced pressure giving the isolated polymer, MA/THFMA 1:1. Polymer molecular weight analysis for all polymers was carried out by GPC/SEC using the DMF solvent at 0.6 mL/min, 40 °C, using polystyrene standards.

### Synthesis of Amide Derivatives of MA/Alkyl Acrylate Copolymers

**Figure 3:**
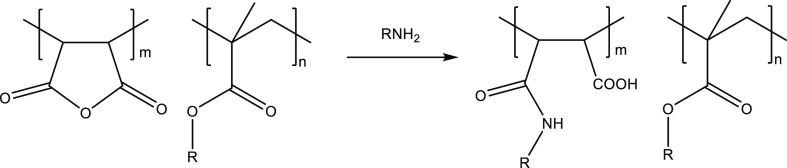
Reaction of MA/alkyl methacrylate polymers with primary amines.

In general, one equivalent of amine was used for each MA monomer unit (Figure 3). The amine and MA polymer were mixed with one or more solvents [e.g., water or *n*-butoxyethanol (nBGE)] in a glass vial. The mixture was stirred at room temperature overnight. Usually, a clear solution was obtained. The maleamide polymer was kept in the solvent carrier at a determined concentration for KHI testing. ^1^H NMR spectroscopic analysis was used to show the complete loss of free amine due to reaction with the anhydride groups.^25,26^ Details of the synthesized polymers can be seen in Table 1. As this is an addition reaction, the molecular weights can be determined as the sum of the molecular weights of the amine and MA polymer.

**Table 1:** Summary In+formation of the Synthesized Maleic-Based Polymers

maleic polymer	*M*_n_ [g/mol]	PDI	solvent carrier
PMA	800	3.8	*o*-xylene
MA/MeAcrylate 1:1	51,000	2.96	*p*-xylene
MA/*i*PrMA 1:1	26,700	3.65	*p*-xylene
MA/*n*BuA 1:1	1400	4.6	*p*-xylene
MA/*n*BuA 1:1	8400	2.8	DME
MA/*n*BuMA 1:1	18,800	1.95	*p*-xylene
MA/*i*BuA 1:1	2900^a^	7.1	*p*-xylene
MA/*i*BuA 1:1	8300	2.65	DME
MA/CHMA 1:1	3600^b^	2.00	DME
MA/THFMA 1:1	2300^b^	1.07	DME

a Minor peak at *M*_n_ = 400–1200 g/mol.

b Peak also seen at a much higher *M*_n_ value but suspected to be aggregated.

### Cloud Point (*T*_Cl_) Measurement

A 2500 ppm aqueous solution of the polymer was heated slowly. The temperature at the first sign of clouding of the solution was taken as the cloud point. Any solution that was already opaque or cloudy at room temperature was first kept in a cooling room at 4 °C before heating. Cloud point measurements were repeated to check reproducibility.

### KHI Performance Tests

Performance testing as KHIs of all polymers was carried out in high-pressure rock cells, which are rocked in a water bath at variable temperatures.^26,27^ The rig (RC5) was supplied by PSL Systemtechnik, Germany. A synthetic natural gas (SNG) blend (Table 2) was used in most as well as a methane/propane 90:10 molar ratio mixture. Both gas blends were made by Yara Praxair, Norway, and the composition was analyzed to be within ±0.1% of all the required concentrations. The equilibrium temperature (*T*_eq_) for sII gas hydrate at 76 bar of SNG was predicted to be 20.5 °C using PVTSim software, Calsep.^28^ For the methane/propane blend, *T*_eq_ is 22.4 °C, according to the literature.^29^

**Table 2:** Composition of the Synthetic Natural Gas (SNG) Mixture

component	mol %
nitrogen	0.11
*n*-butane	0.72
isobutane	1.65
propane	5.00
CO_2_	1.82
ethane	10.3
methane	80.4

Slow constant cooling (SCC) tests were carried out to evaluate the KHI performance of all polymers. This method has been used by our group for many years using the same equipment and SNG which enables us to compare the performance of new KHIs to a plethora of previously tested KHIs.^30^ The standard procedure for SCC tests was as follows:

1. About 105 mL of the KHI solution with dissolved polymer was prepared at least one day before the KHI performance tests to ensure complete dissolution. 20 mL of the KHI solution was added to each cell.
2. The procedure of purging with SNG and then vacuum was applied twice to remove the air in the system.
3. Approximately 76 bars of SNG was loaded to each cell at a temperature of 20.5 °C. The gas inlet/outlet valve of each cell was then turned off, so each cell was a separately closed system.
4. The cells were slowly cooled down at a cooling rate of 1 °C/h and rocked at a rocking rate of 20 full swings/min with a maximum of 40°. The pressure and temperature data during the cooling period were recorded by sensors.

**Figure 4:**
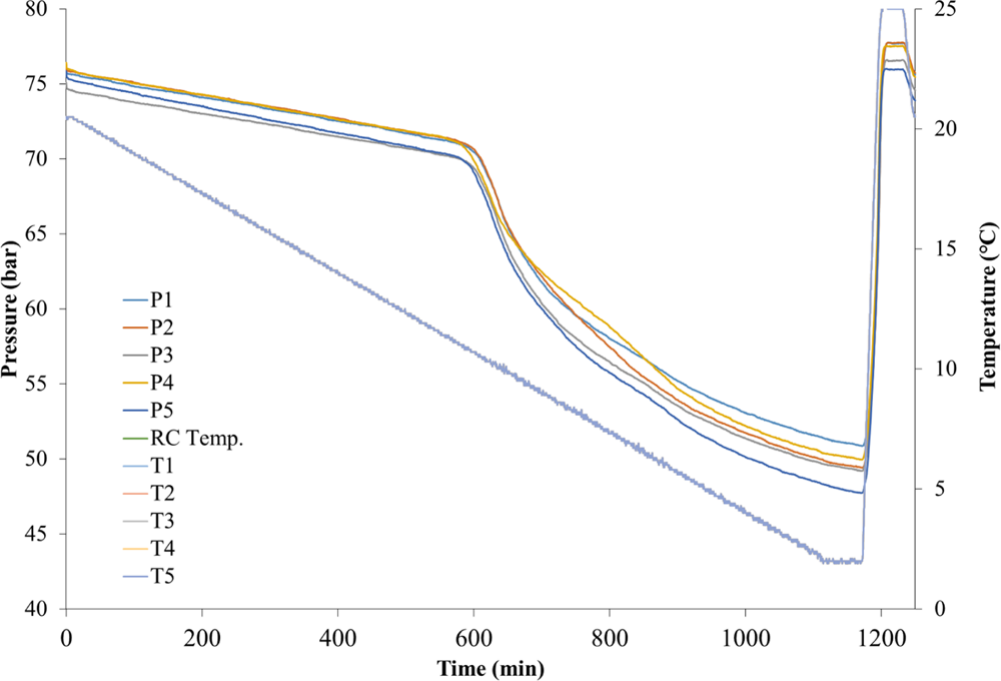
Pressure–time and temperature–time curves obtained from all five cells in steel rocking cell SCC tests.

An example of the pressure–time and temperature–time curves obtained from one set of five parallel rocking cell experiments is shown in Figure 4.

**Figure 5:**
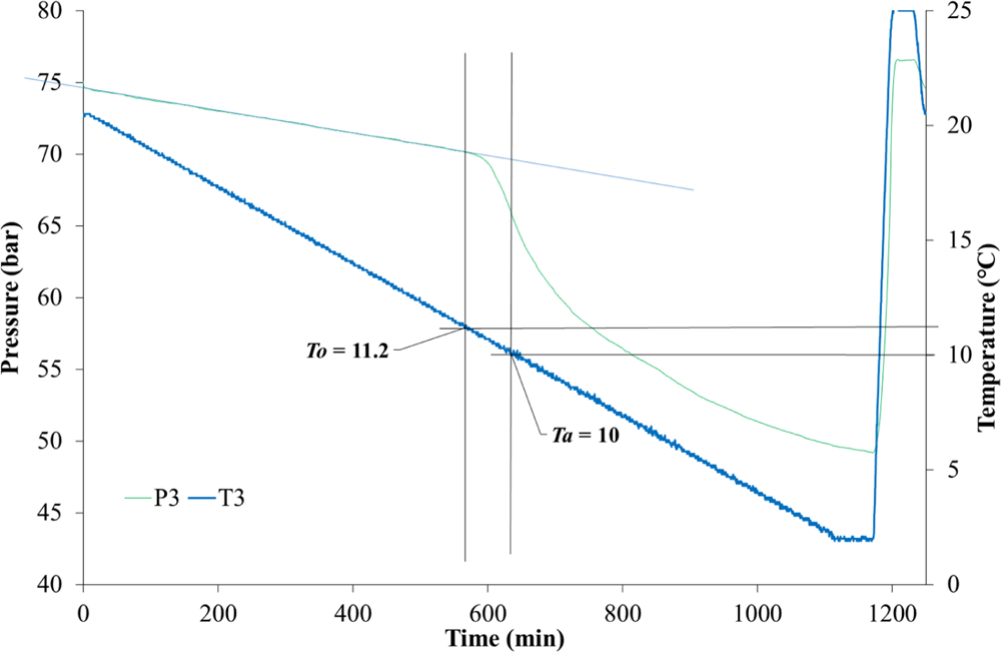
Determination of *T*_o_ and *T*_a_ values in an SCC test in a steel rocking cell.

The determination of hydrate onset temperature (*T*_o_) and rapid hydrate formation temperature (*T*_a_) from the temperature and pressure curves obtained from one cell is shown in Figure 5. In the closed system, the pressure decreased linearly due to the constant cooling of the temperature. Once gas hydrates started to form, the pressure deviated from the original linear track, and this first pressure drop point was marked as *P*_o_. The corresponding temperature at *P*_o_ was determined as *T*_o_. The fastest pressure drop point was marked as *P*_a_, and its corresponding temperature was determined as *T*_a_.

As we have observed in many past studies, the *T*_o_ value varies by a margin of error 10–15%, whereas *T*_a_ values vary by up to 10%.^26,27^ The margin of error generally increases as the average *T*_o_ value for a set of data decreases. Standard deviations for all data sets were also determined, assuming a normal distribution.

## Results and Discussion

Polymerizations were originally conducted in xylene solvent. However, xylene gave a very low-molecular weight polymer, as can be seen for polymaleic anhydride (PMA) in Table 3, so we tried a more polar solvent. We chose DME as it had a high-enough boiling point (85 °C) for the initiation of polymerization by AIBN as well as being unreactive to MA. For the amination of maleic polymers, we used either water or nBGE as a well-known KHI high-flash point solvent synergist. nBGE was also used in the original work on maleic-based KHI polymers in the 1990s.^25^

**Table 3:** Summary of SCC KHI Tests^a^

polymer	NaOH added mol/L	pH before (after testing)	*T*_o_ (av.) [°C]	St. Dev. [°C]	*T*_a_ (av.) [°C]	St. Dev. [°C]	gas
aq HCl		4.5	18.8	0.9	18.2	0.8	SNG
deionized water		6 (5)	16.0	0.6	15.7	0.6	SNG
		6 (6)	18.8	0.3	18.2	0.3	C1/C3
aq NaOH	1.65	>13 (>13)	12.5	0.6	12.3	0.6	SNG
	0.09	13 (13)	18.2	0.3	17.6	0.3	C1/C3
PVCap		7	9.8	0.3	9.4	0.2	SNG
	0.10	13 (13)	4.0	0.5	3.7	0.5	SNG
PMA^b^		5	16.1	0.4	15.9	0.4	SNG
MA/MeA 1:1^b^		5	16.5	0.5	16.1	0.4	SNG
MA/*n*BuA 1:1^b^	1.65	>13 (>13)	6.0	0.9	5.2	0.7	SNG
	0.045	12	15.2	0.7	14.9	0.6	SNG
		4–5	16.0	0.4	15.6	0.3	SNG
MA/*n*BuA 1:1	1.6	>13 (>13)	6.9	2.0	6.3	1.8	SNG
	0.09	13 (8)	14.8	0.2	14.5	0.2	SNG
		2 (2)	15.9	0.4	15.4	0.2	SNG
MA/*i*BuA 1:1^b^		12	12.3	0.1	12.3	0.2	SNG
		4–5	11.3^c^	0.1	11.2	0.1	SNG
MA/*i*BuA 1:1	1.47	>13 (>13)	4.1	0.7	3.4	0.7	SNG
	0.09	13 (13)	14.0	0.3	13.8	0.3	C1/C3
	0.09	13 (8)	13.3	0.1	13.2	0.1	SNG
		2–3 (2–3)	15.2	0.4	14.9	0.3	C1/C3
MA/THFMA 1:1		3–11	insoluble				
MA/cHex 1:1		3–11	insoluble				

a Average of 5 tests with 2500 ppm polymer in water unless otherwise stated.

b Made in xylene.

c 2 tests only.

The results obtained from the SCC tests for MA/alkyl acrylate derivatives are summarized in Table 3. Deionized water and PVCap were also tested for comparison. A concentration of 2500 ppm (0.25 wt %) was chosen as a typical field dosage. Not all pHs were measured after testing, just those that were important for understanding the effect of the added base, as will be discussed below. In general, we use *T*_o_ values to gauge the performance of a KHI as total inhibition of macroscopic hydrate formation is the best to avoid any chance of deposits building up in the flow line. The *T*_a_ values in Table 4 for maleic polymers are all fairly close to the *T*_o_ values (<1 °C), indicating that these polymers do not have a strong effect on preventing macroscopic crystal growth.

**Table 4:** SCC KHI Test Results with SNG for Amide and Ester Derivatives of Maleic Anhydride Polymers

polymer name	*T*_o_ (av.) [°C]	St. Dev. [°C]	*T*_a_ (av.) [°C]	St. Dev. [°C]	*T*_o_ – *T*_a_ (av.) [°C]
no additive	16.0	0.6	15.7	0.6	0.3
PVCap	9.8	0.3	9.4	0.2	0.4
MA/*n*BuA 1:1-MeNH_2_^a^	15.3	0.6	14.8	0.6	0.5
MA/*i*PrA 1:1-DBAPA^a^	11.6	0.1	11.1	0.1	0.5
MA/*i*PrA 1:1-DBAPA-AO^a^	9.8	0.5	9.6	0.5	0.2
MA/*n*BuA 1:1-DBAPA	11.2	0.2	10.9	0.2	0.3
MA/*n*BuA 1:1-DBAPA^a^	12.0	0.6	11.4	0.5	0.6
MA/*n*BuA 1:1-DBAPA-AO	7.1	1.0	6.7	1.0	0.4
MA/*n*BuA 1:1-DBAPA-AO^a^	7.2	0.2	6.9	0.2	0.3
MA/*i*BuA 1:1-DBAPA	11.0	0.2	10.6	0.2	0.4
MA/*i*BuA 1:1-DBAPA-AO	7.1	0.3	7.0	0.3	0.1
MA/CHMA-DBAPA^b^	11.1	0.1	10.8	0.2	0.3
MA/THFMA	insoluble				
MA/THFMA-DBAPA	6.9	0.3	6.1	0.8	0.8
MA/THFMA-DBAPA-AO	6.5	0.1	6.3	0.1	0.2
MA/THF-DBEA	10.9	0.1	10.7	0.2	0.2
MA/THF-DBEA-AO	11.3	0.1	11.2	0.1	0.1

a MA copolymer precursor made in xylene.

b Not fully soluble.

Concerning the maleic polymers, we will discuss the MA/(meth)acrylate copolymers first. This was the original target, to make amphiphilic polymers where the hydrophilicity is provided by the maleic acid groups and the hydrophobic groups provided by a cheap acrylate or methacrylate monomer. Maleic copolymers are usually alternating copolymers due to the low polymerization rate of MA.^31^ We also knew from past research that alkyl groups with about 3–6 carbon atoms in polyamides gave polymers with a good KHI performance.^27,30^ In particular, branching of the tail of the alkyl group or use of a cycloalkyl group is beneficial. Therefore, we began with readily available acrylates and methacrylates with *iso*-propyl, *iso*-butyl, and *n*-butyl groups. We did not use vinyl alkanoates with C3–C4 alkyl groups such as vinyl butanoate (vinyl butyrate) or vinyl pentanoate (vinyl valerate) as their cost is considerably higher than alkyl (meth)acrylates with equivalent size alkyl groups. We chose some methacrylates as we knew the extra methyl group on the backbone has been advantageous for the improved KHI performance of alkylmethacrylamides compared to alkylacrylamides.^32−34^

**Figure 6:**
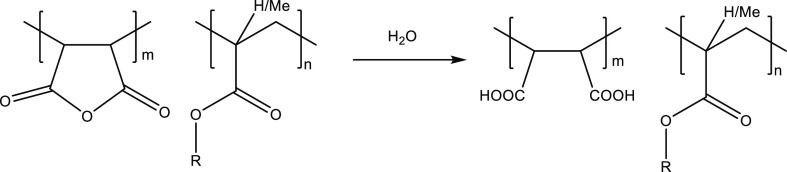
Hydrolysis of MA/alkyl (meth)acrylate copolymers.

The MA units in polymers react slowly (<24 h with stirring) with water to give maleic acid groups (Figure 6). Thus, when making 2500 ppm solutions of PMA and 1:1 MA/Methyl methacrylate copolymer, all MA groups are converted to maleic acid groups before testing. Both these polymers gave a poor KHI performance as expected due to the lack of hydrophobic groups and very small hydrophobic groups, respectively. However, at normal pipeline pH (4–5), the C3–C4 alkyl acrylate 1:1 copolymers, MA/*i*PrMA, MA/*n*BuA, and MA/*i*BuA, all gave a poor KHI performance as well. We encountered some difficulty to dissolve these 1:1 copolymers, so we chose to add a base to see if this would improve the rate and ease of dissolution as well as the performance. (No cloud points were observed for these polymers at any pH). The second column in Table 3 shows the amount of the NaOH base added per 20 mL aq and salts of the polymer solution in each cell. If a sufficient amount was added to neutralize the maleic acid groups, we still obtained poor performance, with average *T*_o_ values of about 15–16 °C. These results suggest that the use of ester and carboxylic acid groups as the hydrophilic parts in the amphiphilic KHI polymer groups is insufficient to give the good KHI good performance.

However, if excess NaOH was added to these MA/alkyl acrylate copolymers such that the pH remained at about 13 after degassing the cells, we obtained much lower *T*_o_ values, from 4 to 6 °C depending on the copolymer (Table 3). The effect of the excess base is at least threefold: first, the acid hydrate-forming gas CO_2_ in the SNG is neutralized affecting polymer–CO_2_ interactions and the reactions kinetics, the pressure is lowered, and the added electrolytes (NaOH, Na_2_CO_3_, or NaHCO_3_) shift the hydrate equilibrium to higher temperatures, giving a lower driving force. To check if this was a more universal effect, we tested the vinyl lactam-based KHI polymer, PVCap. When tested without addition of any base (pH 7), an average *T*_o_ of 9.8 °C was observed, in line with previous studies. When excess NaOH was added, the average *T*_o_ value was 4.0 °C, much lower than without base treatment. This again shows the effect of removing the CO_2_ and lowering the driving force for hydrate formation. The drop in average *T*_o_ value is also seen for the test with just NaOH and no polymer. The average *T*_o_ dropped from 18.8 °C for water, initially at pH 6, to 12.5 °C when 0.066 g NaOH was added to each cell.

As a further study of the effect of CO_2_ removal, we ran tests with a methane/propane 9:1 molar ratio mixture without polymer and with MA/*i*BuA made in DME. With no additives and only a minor amount of NaOH (0.0036 g), enough to keep the pH at 13 before and after the test, there was no significant change in the onset temperature compared to deionized water (average *T*_o_ 18.2 vs 18.8 °C). The same trend was seen with MA/*i*BuA 1:1. We obtained poor KHI performance whether the pH was 2–3 or 13. These results with methane/propane show that the effect of pH alone does not affect the onset temperature, but the amount of base added and the loss of CO_2_ from the SNG are the critical issues.

We also investigated two MA/alkyl methacrylate copolymers, MA/tetrahydrofurfuryl methacrylate 1:1 (MA/THFMA 1:1) and MA/CHMA 1:1 (MA/CHMA 1:1). However, both were insoluble in water at 2500 ppm even when heated, so they were not tested for KHI performance in the rocking cells.

**Figure 7:**
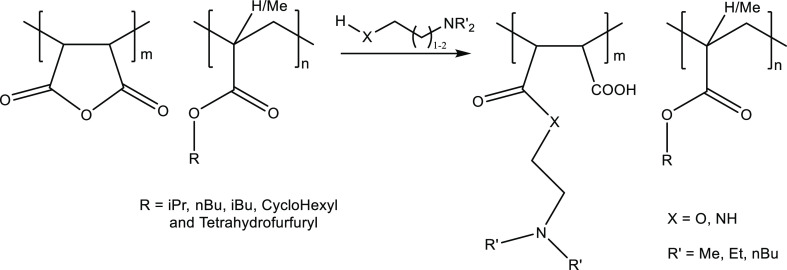
Reaction of MA/alkyl (meth)acrylate copolymers with diamines and alkanolamines.

Because of the poor performance or insolubility of the alkyl (meth)acrylate copolymers, we, therefore, decided to ring-open the anhydride units with diamines or alkanolamines to give pendant dialkylamino groups rather than ring-open in water to give maleic acid groups (Figure 7).

The list of SCC results for amine-derived maleic polymers is given in Table 4. MA precursor polymers were made in DME or xylene. We also knew that nBGE had been used previously to make maleamides from MA copolymers and has been reported to be a good synergist for many KHI series as well as a high-flash-point solvent.^35−37^ Therefore, we used nBGE for the reaction of diamines and alkanolamines with MA/alkyl (meth)acrylate copolymers. All maleic-based copolymers made in nBGE have a polymer concentration of 25.8 wt %. This means any tests at 2500 ppm polymer had additional 7190 ppm nBGE.

We first tried a reaction of the MA units in MA/*n*BuA 1:1 copolymer (made in xylene) with methylamine in nBGE. The polymer was difficult to dissolve but eventually became soluble at 2500 ppm. It gave a poor result with an average *T*_o_ of 15.4 °C. However, this result gives useful evidence that when the amide and the hydrophobic group are not directly covalently bonded to each other, the KHI performance is poor. In common KHI polymers, such as PVCap or PNIPMAM, the hydrophilic and amide groups are directly connected to the side-chain of the same monomer.

Knowing that the hydrophobic group in the alkyl acrylate was probably insufficient for good KHI performance, we introduced more hydrophobic groups by reacting the MA units with a readily available diamine, *N*,*N*-dibutylaminopropylamine (DBAPA). We had used this before with good success to make VA:MA-60%cHex-40%DBAPA, which also showed good corrosion inhibition properties.^26,38^ Also, adding monoamines (alkylamines) to ring-open MA units only lowers the water solubility, so we reasoned this was not a good idea with MA:alkyl acrylate copolymers that are only just water soluble. The results in Table 4 show that MA/*i*PrA 1:1-DBAPA gave some KHI effect with an average *T*_o_ of 11.6 °C. The KHI performance was not significantly different for DBAPA derivatives of larger alkyl acrylates or methacrylates, MA/*n*BuA 1:1-DBAPA, MA/*i*BuA 1:1-DBAPA, and MA/CHMA 1:1-DBAPA. For MA/nBuA 1:1-DBAPA, we made separate polymers using two samples of MA/nBuA 1:1 made in xylene and DME, both of which gave similar results.

Another interesting methacrylate we wished to include in maleic copolymers was THFMA. THFMA contains a pendant tetrahydrofuran ring which on its own is a known sII hydrate former. This makes THFMA a useful group to incorporate into vinylic KHI polymers. Some copolymers with the THFMA monomer polymers have been investigated previously.^39^ These included VCap copolymer and a polyethoxylated methacrylate, which were shown to have good KHI performance.

The synthesized maleic copolymer MA/THMA 1:1 was found to be insoluble in water even when heated. Therefore, this polymer was treated with DBAPA to form MA/THFMA 1:1-DBAPA. This copolymer gave the best KHI performance of the DBAPA-derivatized polymers (not counting their corresponding amine oxides discussed below). We believe the result is due to the pendant tetrahydrofuran rings. MA/THFMA 1:1-DBAPA gave excellent KHI performance with the SNG gas mixture, with an average *T*_o_ of 6.9 °C, about 3 °C lower than PVCap (Table 4). The addition of nBGE probably boosts the performance. The performance of MA/THFMA 1:1-DBAPA was similar to PVCap with a similar amount of added nBGE, which gave an average *T*_o_ of 7.3 °C under the same test conditions in the same rocking cell apparatus.^40^

The precursor copolymer MA/THFMA 1:1 was also reacted with DBEA in nBGE to give MA/THFMA 1:1-DBEA. The average *T*_o_ value for this polymer was 10.9 °C, that is significantly higher than that for MA/THFMA 1:1-DBAPA. We believe this is at least partly due to the ester rather than the amide linkage formed and that hydrogen bonding is stronger for amides.

**Figure 8:**
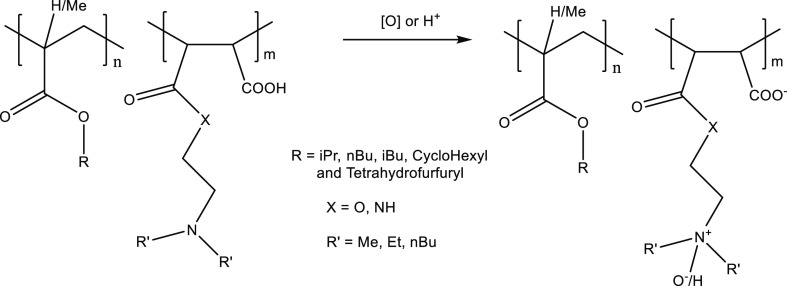
Quaternization or amine oxide formation from the maleic dialkylamino groups.

The good performance of polymers with the dibutylamino end groups (−DBAPA or −DBEA) may also be due to these groups being partly protonated, either by the effect of dissolved acid gas CO_2_ or internally via transfer of a proton from a carboxylic acid group (Figure 8). This quaternization of the dialkylamino groups could give improved performance as we knew from past studies that polymers with pendant butylated quaternary ammonium groups can have good KHI performance.^41−43^

**Figure 9:**
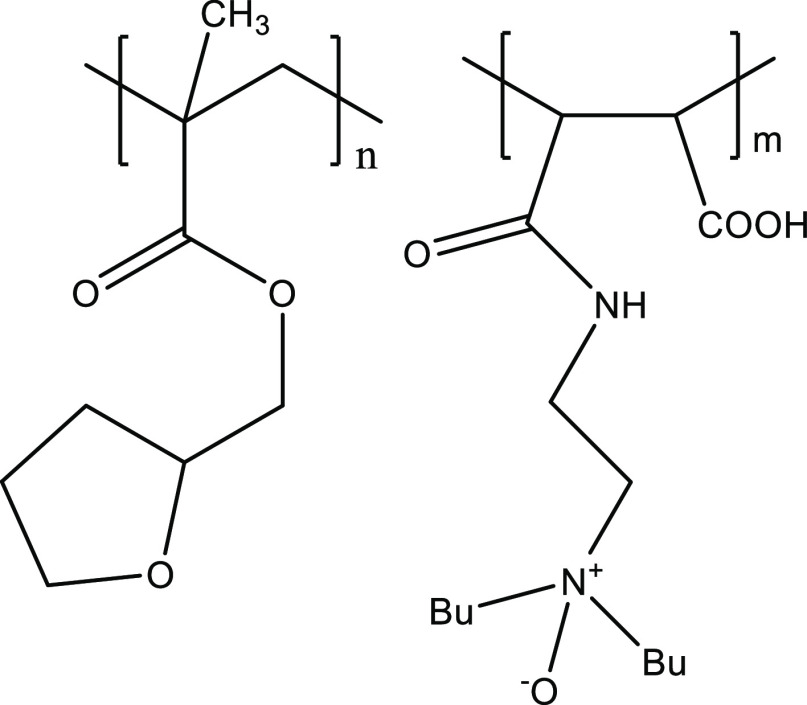
Structure of THFMA:MA-DBAPA-AO terpolymer.

We also knew that amine oxide groups in polymers can give good KHI performance. One study showed that a series of polyamine oxides was significantly better as a KHI than the corresponding polyamines, as well as gave better water solubility.^44^ Therefore, we synthesized several amine oxides of the maleic polymers with dibutylamino head groups by reaction with hydrogen peroxide as previously described (Figure 8).^45^ As the results in Table 4 show, all the maleic-based polyamine oxides gave lower average *T*_o_ values than the equivalent polyamines. The jump in performance is most significant for two butyl acrylate copolymers (*n*- and *iso*-) lowering *T*_o_ by about 4 °C. For MA/THFMA 1:1-DBAPA-AO, there was no significant performance increase compared to the polyamine (Figure 9). This polyamine already had a better KHI performance than the alkylacrylate copolymers. It is possible that the THF ring is attacked by the hydrogen peroxide either to form a peroxide or is ring-opened, giving a less KHI-active monomer.^46,47^ The good KHI performance of MA/THFMA 1:1-DBAPA-AO may then be due to dibutylamine oxide groups. The amine oxide MA/CHMA 1:1-DBAPA-AO was not investigated as the amine MA/CHMA 1:1-DBAPA performed much worse (*T*_o_ = 11.1 °C) than MA/THFMA 1.1-DBAPA (*T*_o_ = 6.9 °C). Thus, we assume that the amine oxide derivative MA/CHMA 1:1-DBAPA-AO will not be better than MA/THFMA 1:1-DBAPA-AO.

The difference between *T*_o_ and *T*_a_ values can give some indication of the ability of a KHI to arrest crystal growth. The average *T*_o_ – *T*_a_ values in Table 4 are all relatively low, suggesting that neither the polyamines nor polyamine oxides have a strong ability to slow crystal growth once nucleation has been detected.

## Conclusions

Maleic acid/alkyl (meth)acrylate (MA/RMA) 1:1 copolymers with varying size and shape hydrophobic groups have been synthesized. At high pH, it is the loss of CO_2_ from the gas phase and the decrease in *T*_eq_ that contribute to the good performance. However, none of the polymers gave good KHI performance at pipeline pH (4–5) despite having good water solubility and optimal size hydrophobic groups. This means that it is the hydrophilic groups (carboxylic acid and ester) that need replacing. Better KHI polymers are found with amide and amine oxide groups which contain nitrogen atoms and have strong hydrogen bonding abilities.

The KHI performance of these MA/alkyl acrylate copolymers was significantly improved by adding pendant dibutylamino groups by reaction with DBAPA or DBEA. Partial quaternization by protonation in acid conditions probably contributes to the good KHI performance. The most effective polymer was MA/THFMA 1:1-DBAPA giving better performance than PVCap. The tetrahydrofuran rings contribute to the performance as shown by the poorer performance of equivalent MA/alkylacrylate copolymers with C3–C4 alkyl groups. Oxidation of the pendant dibutylamino groups in maleic-DBAPA units to form the amine oxide improved the performance further as well as the water solubility. None of the polymers gave a strong ability to arrest the crystal growth once nucleation had been detected.
